# Mediating Role of Depression Severity in the Relationship Between Childhood Trauma and Non‐Suicidal Self‐Injury Among Adolescents With Mood Disorders

**DOI:** 10.1002/brb3.70533

**Published:** 2025-05-13

**Authors:** Jianbing Li, Jiaqi Wang, Changhe Fan

**Affiliations:** ^1^ Department of Psychiatry The Affiliated Guangdong Second Provincial General Hospital of Jinan University Guangzhou China; ^2^ The First Affiliated Hospital of Guangdong Pharmaceutical University Guangzhou China

**Keywords:** adolescents | childhood trauma (CT) | depression | non‐suicidal self‐injury (NSSI)

## Abstract

**Objective:**

Non‐suicidal self‐injury (NSSI) frequently co‐occurs with childhood trauma (CT) and depression; however, their interplay and underlying mechanisms are not fully understood. This article explores the nuanced relationships between these factors and their impact on NSSI, offering practical insights for the prevention and intervention of childhood post‐traumatic depression progression and NSSI among adolescents.

**Methods:**

The present study leveraged the Childhood Trauma Questionnaire (CTQ), the Adolescent Non‐suicidal Self‐injury Assessment Questionnaire (ANSAQ), and the Hamilton Rating Scale for Depression (HAMD) to assess a cohort of 361 individuals with mood disorders. The study design was cross‐sectional, and a convenience sampling method was utilized. The mediation effect was tested using the Process stepwise regression method, and the significance of the mediation effect was assessed through Bootstrap analysis.

**Results:**

Among 361 adolescents with mood disorders, 232 (64.3%) exhibited NSSI behaviors. Analyses revealed that CT was directly associated with NSSI (*β* = 0.312, *p* < 0.001) and indirectly associated through mediation pathways (*β* = 0.256, *p* < 0.001). CT also showed direct association with depression severity (*β* = 0.400, *p* < 0.001). Depression severity was significantly associated with NSSI (*β* =  0.184, *p* = 0.042), suggesting a partial mediating role of depression severity in this relationship.

**Conclusions:**

CT shows a significant association with NSSI among adolescents with mood disorders, with depression severity serving as a partial mediator in this relationship. Active coping strategies targeting depressive symptoms in individuals with a history of CT may help reduce NSSI behaviors.

## Graphical abstract

Among adolescents with mood disorders, childhood trauma significantly impacts NSSI, mediated partially by depression. Addressing depression may reduce NSSI behaviors.

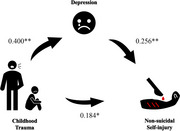

**Abbreviations**
ANSAQ: Adolescent Non‐suicidal Self‐injury Assessment Questionnaire
CCP: child–parent Psychotherapy
CT: childhood trauma
CTQ: Childhood Trauma Questionnaire
DSM‐IV: Diagnostic and Statistical Manual of Mental Disorders
EA: emotional abuse
EN: emotional neglect
HAMD: Hamilton Rating Scale for Depression
ICD‐10: International Classification of Diseases
MDD: major depressive disorder
NSSI: non‐suicidal self‐injury
PA: childhood physical abuse
PN: physical neglect
SA: sexual abuse
SI: suicidal ideation

## 1 Introduction

Non‐suicidal self‐injury (NSSI), referring to intentionally causing harm to oneself without the intention of dying, is a widespread mental health problem that is particularly prevalent among adolescents (Bae et al. 2020). Typically, it emerges in early adolescence and peaks during mid‐adolescence (Plener et al. 2015). A global meta‐analysis revealed that the worldwide prevalent occurrence of NSSI among children and teenagers is 19.5% (Lim et al. 2019). NSSI behavior has emerged as a significant concern, particularly among adolescents, who experience the highest incidence, making it a prominent global health challenge for this age group (Jacobson and Gould 2007).

Childhood trauma (CT) refers to recurrent adverse experiences that deviate from the anticipated environment and demand adaptation (Turecki et al. 2014; Merrick et al. 2019). Illustrations encompass physical, sexual, and emotional maltreatment, parental illness, criminality, violence, negligence, and poverty (McLaughlin et al. 2012; Krugers et al. 2017). The long‐term repercussions of CT can manifest as detrimental impacts on a child's cognition, mood, and behavior, leading to mental health issues, social problems, and physical health challenges (López‐Martínez et al. 2018; Laursen and Collins 1994; Terr 1991). CT has a global reach, causing long‐lasting physical and psychological wounds to children and placing significant emotional and economic strains on parents, schools, and society as a whole (De Bellis and Zisk 2014).

Depression, recognized formally as major depressive disorder (MDD), is a prevalent and significant mood disorder impacting cognitive processes, emotions, and health (Cui 2015). It is marked by persistent sadness, lethargy, and a diminished capacity to experience pleasure (Liu et al. 2023). Over the last three decades, there has been an almost 50% rise in global cases, with over 264 million individuals of various ages now affected (Liu et al. 2020). The World Health Organization identifies depression as the foremost cause of mental and physical incapacity globally and a significant factor in the worldwide disease burden (Monroe and Harkness 2022).

NSSI is a common issue among teenagers, shaped by a variety of influences such as social integration challenges, interpersonal pressures, underlying neurobiological factors, emotional regulation difficulties, and the impact of trauma experiences during childhood (Brown and Plener 2017; Wang et al. 2022). Extensive research conducted at both national and international levels has consistently demonstrated that traumatic events during childhood serve as a distinct and influential risk factor for engaging in NSSI behavior (Wan et al. 2015; Liu et al. 2018). Empirical data indicate a strong and positive link between CT experiences and the occurrence or frequency of NSSI in adolescents (Brunstein Klomek et al. 2016). Additionally, CT has been confirmed as a distinct factor within the framework that explains the occurrence of NSSI as the dependent variable (Xie et al. 2023).

NSSI is frequently observed in the context of depression. Among the various risk factors linked to NSSI, depressive and anxious symptoms are notably prevalent mental health conditions (Wang et al. 2022). The interplay between depression and NSSI has garnered considerable attention, with numerous studies indicating a positive correlation between the two (Hankin and Abela 2011). Confronted with the overwhelming negative emotions and emotional numbness that characterize depression, individuals may turn to self‐harm as a coping mechanism (Marshall et al. 2013).

Furthermore, there is a large research literature supporting the link between CT and depression (Nikkheslat et al. 2020; Xie et al. 2018). Research has indicated that CT can influence the development of depression via the mediating mechanism of cognitive emotion regulation strategies (CERS) (Chu et al. 2022). Different kinds of CTs have been demonstrated to be related to the onset, severity, and progression of depressive symptoms (Gamble et al. 2006; Gibb et al. 2007; van Veen et al. 2013). Depressed patients who have endured CT are more inclined to attempt suicide (Fuller‐Thomson et al. 2016; Schönfelder et al. 2019). Depression, which is widespread among adolescents, is regarded as a crucial risk factor for both NSSI and sexual abuse (SA) (Jiao et al. 2022; He et al. 2023).

However, the way in which CT, depression severity, and NSSI interact with one another remains unclear. Psychodynamic theory, an early theoretical model for NSSI, aims to uncover the connection between NSSI behaviors and desires, particularly in the context of child abuse and mental disorders (Levy et al. 2007). This theory posits that childhood experiences lay the foundation for adult personality and interpersonal relationships, emphasizing the significance of unconscious drives in human functioning (Westen 1998). Psychodynamic theory delves into how internal psychological drives shape human behavior and personality (Teater 2015). It asserts that much of our mental life is unconscious, with our adult behaviors and feelings rooted in childhood experiences (Briggs et al. 2019). This theoretical framework is based on the core belief that past experiences, especially traumatic ones, play a pivotal role in shaping current behaviors (Pitman and Knauss 2020).

Building upon these theoretical foundations, we hypothesize that depression severity mediates the association between CT and NSSI. This proposed mediation model indicates a sequential association where CT shows a significant relationship with elevated depression severity, which in turn demonstrates a consistent linkage with increased NSSI engagement. These findings underscore the clinical importance of understanding these pathways to inform targeted prevention and intervention strategies for co‐occurring mood disorders and NSSI.

## 2 Materials and Methods

### 2.1 Study Population

From May 2022 to August 2024, a convenience sampling approach was used to recruit 380 individuals diagnosed with mood disorders from both the outpatient and inpatient psychiatry units at Guangdong Second Provincial General Hospital.

### 2.2 Measurements

The Childhood Trauma Questionnaire (CTQ) is a widely recognized and standardized instrument for evaluating the spectrum (Bernstein et al. 2003). This assessment comprises 28 items that measure five distinct dimensions: physical abuse (PA), emotional abuse (EA), SA, physical neglect (PN), and emotional neglect (EN). Each item is typically rated using a 5‐point scale, ranging from “never” to “very often.” The CTQ is a valuable tool in both clinical practice and research, enabling professionals to gauge the profound effects of CT on an individual's psychological health, mental well‐being, and social interactions.

The Adolescent Non‐suicidal Self‐injury Assessment Questionnaire (ANSAQ) is a standardized assessment tool designed to evaluate NSSI behaviors in adolescents (Wan et al. 2018). It consists of 12 items that are categorized into 2 factors: self‐injury behavior without evident tissue damage and self‐injury behavior with evident tissue damage. The questionnaire aims to measure the frequency of NSSI behaviors that adolescents engage in, providing insights into the presence and severity of self‐injury. We identified the NSSI group through admission interviews, classifying individuals as part of the NSSI group if they exhibited any form of self‐harm, regardless of whether it involved tissue damage.

The Hamilton Rating Scale for Depression (HAMD) is a highly regarded and extensively utilized instrument for gauging the intensity of depressive symptoms. HAMD typically contains 17–21 items, each of which is rated from 0 to 4 based on the severity of the symptom. We employed the 17‐item Hamilton Depression Rating Scale (HAMD‐17) to assess core depressive symptoms, this version being the internationally standardized instrument for evaluating depression severity (Hamilton 1960). It encompasses an evaluation of various indicators such as a depressed mood, feelings of guilt, disruptions in sleep patterns, and alterations in appetite or weight. Medical professionals or researchers assign scores to each criterion, relying on either self‐reported information or observed conduct. The aggregate score derived from this scale serves as an indicator of the comprehensive severity of depression, with higher scores signifying a greater degree of symptomatic distress.

Data were gathered through the administration of paper‐based survey questionnaires, which were designed to be completed within approximately 1 h. Throughout the process, participants were assured that researchers would be available to patiently answer any queries or address concerns related to the survey. Participants were encouraged to complete the survey in a quiet and comfortable setting. The collected data were then meticulously reviewed and cross‐checked by the research team. In instances where there were noticeable omissions or inconsistencies, participants were requested to correct or complete the information immediately. Following the initial data collection, a thorough double‐check was conducted to ensure accuracy and completeness.

Inclusion criteria: (A) Han Chinese ethnicity, aged 12–18, any gender. (B) Met diagnostic criteria for mood disorders according to the 10th edition of the International Classification of Diseases (ICD‐10) and the 4th edition of the Diagnostic and Statistical Manual of Mental Disorders (DSM‐IV), either as first onset or recurrent episodes (Sheehan et al. 1998). (C) At least one of the following mood episodes was present at the time of inclusion and had not achieved clinical remission: depressive episode, mild depressive episode, hypomanic episode, manic episode, mixed episode, or persistent mood dysregulation, significantly impacting social functioning or causing significant distress (for retrospective surveys, individuals who had achieved clinical remission were included). (D) The diagnosis of mood disorders must be confirmed by two psychiatrists with a senior professional title within the research team.

Exclusion criteria: (A) Presence of neurological disorders or other severe physical illnesses. (B) Mood episodes secondary to organic mental disorders, substance use, severe physical illnesses, or other functional psychiatric disorders. (C) Severe, persistent, and intense suicidal ideation (SI) or behavior, or severe and persistent disruptive or impulsive behavior, with an unwillingness or inability to receive psychological intervention or psychoeducation. *Please note that criterion does not apply to retrospective surveys and does not exclude individuals with intermittent, non‐intense suicidal tendencies.

### 2.3 Patient and Public Involvement

The participants had no role in the active planning, implementation, reporting, or dissemination of the research. Additionally, there are currently no intentions to convey the study's results back to the participants.

### 2.4 Statistical Analysis

We conducted data analysis using SPSS 26.0 and PROCESS v4.1 software (author: Andrew F. Hayes). For quantitative variables that followed a normal distribution, we described them using the mean ± standard deviation and conducted statistical analyses using *t*‐tests and Pearson correlation analysis. For quantitative variables that did not follow a normal distribution (as confirmed by the Shapiro–Wilk test), we described them using medians and performed nonparametric analyses, including the chi‐square test and Spearman's rank correlation (to assess associations between variables). We selected the Process method of stepwise regression for structural equation modeling (SEM) analysis. In this model analysis, the independent variable was CT, the dependent variable was NSSI, and the mediating variable was depression severity. Among these, NSSI was treated as a continuous variable (based on the total score of ANSAQ), and CT was modeled using its total score. Finally, we used the Bootstrap method to correct for bias and tested the mediating effect on 5000 resampled datasets, calculating direct, indirect, and total effects. The ratio of the mediating effect was the total indirect effect divided by the total effect. A two‐tailed *p* value less than 0.05 was considered statistically significant. In this study, we opted for stepwise regression rather than SEM, primarily due to the relatively small sample size (*n* = 361) and the low complexity of the model. Stepwise regression provides a more intuitive approach to examining the sequential pathways of the mediation effects, whereas the Bootstrap method has effectively validated the robustness of these mediation effects.

### 2.5 Ethics Statement

Ethical clearance for this study was granted under the project title “Research on Clinical Characteristics and Early Identification and Comprehensive Intervention Techniques of Adolescent Mood Disorders,” with the reference number 2024‐KY‐KZ‐014. Before engaging in the study, all participants were required to provide their informed consent, signifying their voluntary agreement to take part in the research.

## 3 Results

### 3.1 Sample Characteristics and Preliminary Analyses

In this study, 380 questionnaires were initially distributed, and we successfully retrieved and validated 361 of them, achieving a robust response rate of 95.0%. The study sample comprised 361 adolescents diagnosed with mood disorders, with an average age of 15.67 years and an SD of 1.89 years. The gender distribution was 109 boys (30.2%) and 252 girls (69.8%). Table 1 illustrates the differences in age, gender, HAMD scores, and experiences of CT among participants with and without NSSI. The analysis indicates that there are no differences between the two groups in terms of gender, age, and SA. However, significant differences were observed in other types of CT, specifically EA (*p* = 0.000), PA (*p* = 0.000), EN (*p* = 0.047), and PN (*p* = 0.043), as well as in HAMD scores (*p* = 0.000). This indicates that individuals in the NSSI group, compared to the non‐NSSI group, exhibit higher depression scores and show a greater association with various forms of CT.

**TABLE 1:** Comparison of general information and questionnaires between non‐suicidal self‐injury (NSSI) and non‐NSSI groups (*n* = 361).

Variables	NSSI (*n *= 252)	Non‐NSSI (*n *= 129)	*t*/χ^2^	*p*
Age	15.48 ± 2.18	15.86 ± 1.77	−1.681	0.075
Gender			1.459	0.227
Female	167 (71.98%)	85 (65.89%)		
Male	65 (28.02%)	44 (34.11%)		
HAMD	22.62 ± 14.21	14.01 ± 9.80	6.116	0.000**
Childhood trauma				
EA	11.43 ± 5.35	6.47 ± 4.93	8.665	0.000**
PA	7.54 ± 4.14	4.93 ± 3.43	6.087	0.000**
SA	6.14 ± 2.92	4.48 ± 2.71	5.315	0.646
EN	13.42 ± 5.20	10.43 ± 6.53	4.770	0.047*
PN	9.75 ± 3.10	6.58 ± 3.94	8.418	0.043*

Abbreviations: EA, emotional abuse; EN, emotional neglect; HAMD, Hamilton Rating Scale for Depression; PA, physical abuse; PN, physical neglect; SA, sexual abuse.

*p < 0.05.

**p < 0.01.

### 3.2 Correlation Analysis of Major Study Variables

We conducted correlation analyses between CT, NSSI, and HAMD scores (Table 2). The scores for D1 and D2 did not follow a normal distribution (Shapiro–Wilk test, *p* < 0.05); therefore, Spearman's correlation analysis was used. D1 scores ranged from 1 to 15 with a median of 8 (IQR: 5–11); D2 scores ranged from 0 to 9 with a median of 3 (IQR: 1–5). The findings indicate that EA, PA, and SA are substantially and advantageously correlated with the first dimension of NSSI, which pertains to acts without tissue damage (D1), and the second dimension, which involves acts with tissue damage (D2). This suggests that the primary forms of CT associated with NSSI may be EA, PA, and SA. A significant positive correlation was observed between these three types of CT and depression severity, indicating that they may also be key factors associated with depressive symptoms. Additionally, both D1 and D2 of NSSI are significantly and positively correlated with depression severity, highlighting a strong association between NSSI—regardless of tissue damage—and the severity of depressive symptoms.

**TABLE 2:** Correlation analysis between childhood trauma, non‐suicidal self‐injury (NSSI), and Hamilton Rating Scale for Depression (HAMD) (*r*) (*N* = 361).

Variables	EA	PA	SA	EN	PN	D1	D2
PA	0.645^**^						
SA	0.396^**^	0.392^**^					
EN	−0.256^**^	−0.1821^**^	−0.032				
PN	0.217^**^	0.270^**^	0.130^**^	0.387^**^			
D1	0.370^**^	0.355^**^	0.270^**^	−0.091	0.290^**^		
D2	0.237^**^	0.301^**^	0.213^**^	−0.116	0.120	0.554^**^	
HAMD	0.430 ^**^	0.224^**^	0.218^**^	−0.059	0.051	0.224^**^	0.157^**^

Abbreviations: D1: dimensions 1 of NSSI; D2: dimensions 2 of NSSI; EA, emotional abuse; EN, emotional neglect; PA, physical abuse; PN, physical neglect; SA, sexual abuse.

*p < 0.05.

**p < 0.01.

### 3.3 Mediation Analysis of CT, Depression Severity, and NSSI

We have constructed a mediation model to examine the significant associations among CT, depression severity, and NSSI, as depicted in Table 3. The mediation analysis was conducted using stepwise regression, yielding the following results: In Model 1, CT showed a significant association with NSSI (*β* = 0.312, *p* < 0.001), establishing the total effect. Model 2 demonstrated a significant relationship between CT and depression severity (*β* = 0.400, *p* < 0.001). Model 3 revealed that CT maintained a significant link with NSSI (*β* = 0.256, *p* < 0.001), whereas depression severity was significantly related to NSSI (*β* = 0.184, *p* = 0.042). These findings indicate that depression severity partially mediates the association between CT and NSSI. This analysis highlights the complex interrelationships among CT, depression severity, and NSSI, offering valuable insights into potential mechanisms underlying NSSI development.

**TABLE 3:** Process mediation effect test results.

Model	Model 1			Model 2			Model 3		
Dependent variable	NSSI			Depression			NSSI		
Indicators	*β*	*t*	*p*	*β*	*t*	*p*	*β*	*t*	*p*
Childhood trauma	0.312	4.975^**^	0.000	0.400	6.611^**^	0.000	0.256	3.776^**^	0.000
Depression							0.184	4.467^*^	0.042
*R^2^*	0.097			0.160			0.113		
Adjusted *R^2^*	0.093			0.156			0.106		
*F*	24.751^**^		0.000	43.704^**^		0.000	14.632^**^		0.000

Abbreviation: NSSI, non‐suicidal self‐injury.

*p < 0.05.

**p < 0.01.

**FIGURE 1:**
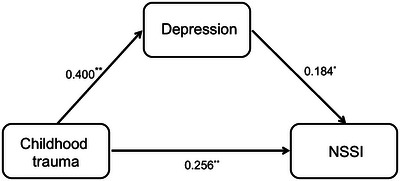
The role of depression as a mediator in the relationship between childhood trauma and non‐suicidal self‐injury. *p < 0.05; **p < 0.01.

The significance of the mediation effect was rigorously assessed using the Bootstrap method, with a robust sampling of 5000 repetitions to calculate the 95% confidence interval. The findings are presented in Table 4. The 95% confidence interval for the mediation effect spans from 0.0017 to 0.0282, which does not include zero. This exclusion of zero in the interval confirms the establishment of an indirect effect, indicating that depression severity significantly mediates the relationship in the model. Furthermore, after controlling for the mediating variables, the confidence interval for the direct effect of the independent variable, CT, on NSSI also excludes zero. This finding suggests that the partial mediation effect of depression severity is indeed significant. The mediation effect value stands at 0.0144, which represents 17.78% of the total effect value of 0.0810. The mediating role of depression severity between CT and NSSI is visually depicted in Figure 1, providing a clear illustration of this complex relationship.

**TABLE 4:** Bootstrap mediation effect test results.

Effect relationship	Effect size	LLCI	ULCI	Proportion of effect (%)
Total effect	0.0810	0.0318	0.1013	
Direct effect	0.0666	0.0002	0.0318	82.22
Mediation effect	0.0144	0.0017	0.0282	17.78

Abbreviations: LLCI, lower limit of confidence interval; ULCI, upper limit of confidence interval.

## 4 Discussion

The research results show that depression severity acts as a mediator in the connection between CT and NSSI, highlighting an indirect pathway. Adolescence is a pivotal time to implement preventive strategies and interventions aimed at self‐harm behaviors. NSSI is notably prevalent among adolescents in non‐clinical settings, with a marked escalation in its prevalence (Xiao et al. 2022). Such behaviors during this developmental stage can have lasting and significant effects, potentially leading to later‐life issues such as anxiety, depression, and SI, as well as imposing additional social and familial strains (Gulbas et al. 2015). Consistent with previous studies, CT is significantly associated with NSSI in adolescents. Negative life events and coping mechanisms act as potential mediators (Xie et al. 2023; Huang et al. 2022). A cross‐sectional examination of CT and NSSI revealed that of the 311 adolescent patients exhibiting NSSI behaviors, 250 (80.39%) had experienced trauma during their childhood (Cheng et al. 2023).

Numerous studies have consistently demonstrated that CT is a pervasive and potent risk factor for a range of psychological disorders, including SI and suicidal behavior (Schönfelder et al. 2019; Norman et al. 2012; Afifi et al. 2008). Traumatic experiences in childhood, such as EA, PA, SA, EN, and PN, are well‐documented contributors to the development of depression (Wen et al. 2021). The German LAC Depression Study underscored the significance of this link, revealing that a staggering 75.6% of individuals with chronic depression had a history of clinically significant CT. Notably, 37% of these patients reported having endured multiple forms of CT, which was found to be correlated with a markedly increased severity of depressive symptoms (Negele et al. 2015; Huh et al. 2014). Moreover, experiencing specific types of early life traumas, particularly physical, emotional, and SA, before the age of 7, can have an impact on the subsequent reaction to antidepressant treatment for MDD (Williams et al. 2016).

Extensive research has consistently established a robust link between depressive mood states and NSSI, with depression emerging as one of the key factors associated with NSSI (Plener et al. 2015; Huang et al. 2021). A longitudinal study examining the interplay between depression and NSSI has shown that depressive symptoms are predictive of future escalations in NSSI behaviors and has also underscored a significant correlation between these symptoms and NSSI (Marshall et al. 2013). Adolescents often resort to NSSI as a strategy to manage their depressive symptoms. Interestingly, the act of NSSI can lead to a temporary alleviation of negative emotions, which in turn may inadvertently reinforce the use of NSSI as a coping mechanism for dealing with depressive symptoms (Dierker et al. 2007; Nock and Prinstein 2004; Briere and Gil 1998; Nock 2009). This cycle underscores the complexity of the relationship between depression severity and NSSI in the adolescent population.

Our research discoveries emphasize the mediating role of depression severity in the link between CT and NSSI and stress the necessity of addressing depressive symptoms as a preventive step against NSSI. Concurrently, it is essential to focus on mitigating the impact of CT by enhancing child–parent psychotherapy (CCP). This can be achieved by fostering a nurturing, secure, and affectionate parent–child dynamic, thereby reducing the likelihood of traumatic experiences in early life (Lieberman et al. 2005). For adolescents suffering from depression who have a history of CT, vigilant monitoring of NSSI behaviors is paramount. It is crucial to devise personalized therapeutic and supportive strategies tailored to their specific needs, ensuring a comprehensive approach to their mental health care.

## 5 Limitations

This research possesses several limitations that should be acknowledged. First, although this study employed assessment tools known for their reliability and validity, they relied solely on self‐evaluations. Moreover, given the absence of an authoritative self‐rating scale for depression tailored specifically to minors, we were compelled to utilize the HAMD scale, which is designed for adult populations. It is crucial to be aware that self‐reported data can be affected by social expectations, recall bias, and response bias, which may result in a possible absence of objectivity. Including additional sources of information, such as interviews, expert judgments, or medical records, could provide a more comprehensive understanding of the phenomenon.

Second, the data collection for this study was conducted using convenient sampling exclusively from a hospital in Guangzhou, China. This sampling method might limit the applicability of the conclusions to a more extensive population. Furthermore, the ideal sample size for survey‐based studies is typically 5–10 times the number of variables in the questionnaire, and our sample size was slightly insufficient. Moving forward, we intend to gather additional data and use a larger sample size to reinforce the validity of our findings. As a result, the outcomes warrant additional scrutiny, and it is necessary to be cautious when generalizing the findings to different settings or populations.

Third, this study employed a cross‐sectional survey design, which limits the ability to examine temporal dynamics among the investigated variables. As a result, we cannot establish a clear temporal sequence among CT, depression severity, and NSSI. Moreover, it is well‐documented that NSSI behaviors in adolescents with depression are dynamic and may change over time. Therefore, future studies should adopt longitudinal designs to track the temporal relationships among CT, depression severity, and the onset of NSSI. This approach would enable a more accurate determination of whether traumatic experiences indeed precede NSSI behaviors and provide stronger evidence for causal interpretations, thereby enhancing our understanding of the progression and interactions of these behaviors and their associated factors.

In summary, although this study provides valuable perspectives, it is crucial to recognize these limitations and encourage more research to tackle them, thereby improving the robustness and applicability of the results.

## 6 Conclusion

Adolescents with severe depressive states, mood disorders, and NSSI frequently present with comorbid CT. The findings of this study indicate that both CT and depression severity show significant associations with NSSI, exerting indirect effects through a chain mediation pathway. These results underscore the importance of psychosocial factors in the prevention and intervention of NSSI behaviors. To further our understanding and enhance the support for individuals at risk, it is imperative to delve deeper into the protective mechanisms that may counteract NSSI. This will enable the development of psychological interventions that are reliable, effective, and readily accessible to patients in need.

## Author Contributions

**Jianbing Li**: conceptualization, writing–original draft, software. **Jiaqi Wang**: writing–original draft, writing–review and editing, data curation. **Changhe Fan**: supervision, funding acquisition, project administration.

## Conflicts of Interest

The authors declare no conflicts of interest.

### Peer Review

The peer review history for this article is available at https://publons.com/publon/10.1002/brb3.70533

## Data Availability

The datasets utilized and analyzed in the current research are not available for public access due to ethical restrictions related to patient data and privacy. Nevertheless, these datasets can be accessed by contacting the corresponding author, who will consider requests for access in a manner that respects the conditions of ethical approval.
